# Exploring librarians' practices when teaching advanced searching for knowledge synthesis: results from an online survey

**DOI:** 10.5195/jmla.2024.1870

**Published:** 2024-07-01

**Authors:** Glyneva Bradley-Ridout, Robin Parker, Lindsey Sikora, Andrea Quaiattini, Kaitlin Fuller, Margaret Nevison, Erica Nekolaichuk

**Affiliations:** 1 glyneva.bradley.ridout@utoronto.ca, Liaison & Education Librarian, Gerstein Science Information Centre, University of Toronto, Toronto, ON; 2 robin.parker@dal.ca, Evidence Synthesis Librarian, WK Kellogg Health Sciences Library, Dalhousie University, Halifax, NS; 3 lindsey.sikora@uottawa.ca, Head (Health Sciences, Medicine and STEM), Health Sciences Library, University of Ottawa, Ottawa, ON; 4 andrea.quaiattini@mcgill.ca, Liaison Librarian, Schulich Library of Physical Sciences, Life Sciences, and Engineering, McGill University, Montreal, QC; 5 kfuller@stfx.ca, Scholarly Communications & Health Sciences Librarian, Angus L. Macdonald Library, St. Francis Xavier University, Antigonish, NS; 6 m.nevison@mail.utoronto.ca, Graduate Student Library Assistant, Gerstein Science Information Centre, University of Toronto, Toronto, ON; 7 erica.lenton@utoronto.ca, Faculty Liaison & Education Librarian, Gerstein Science Information Centre, University of Toronto, Toronto, ON

**Keywords:** Evidence Synthesis, teaching strategies, Literature Searching

## Abstract

**Objective:**

There is little research available regarding the instructional practices of librarians who support students completing knowledge synthesis projects. This study addresses this research gap by identifying the topics taught, approaches, and resources that academic health sciences librarians employ when teaching students how to conduct comprehensive searches for knowledge synthesis projects in group settings.

**Methods:**

This study applies an exploratory-descriptive design using online survey data collection. The final survey instrument included 31 open, closed, and frequency-style questions.

**Results:**

The survey received responses from 114 participants, 74 of whom met the target population. Some key results include shared motivations to teach in groups, including student learning and curriculum requirements, as well as popular types of instruction such as single session seminars, and teaching techniques, such as lectures and live demos.

**Conclusion:**

This research demonstrates the scope and coverage of librarian-led training in the knowledge synthesis research landscape. Although searching related topics such as Boolean logic were the most frequent, librarians report teaching throughout the review process like methods and reporting. Live demos and lectures were the most reported approaches to teaching, whereas gamification or student-driven learning were used rarely. Our results suggest that librarian's application of formal pedagogical approaches while teaching knowledge synthesis may be under-utilized, as most respondents did not report using any formal instructional framework.

## INTRODUCTION

In health sciences, early career researchers and students are frequently encouraged to conduct knowledge synthesis (KS) reviews to situate their research program in the context of what has previously been done, to gain an understanding of the research process, to increase critical appraisal skills, and to fulfill academic requirements [1–3]. While narrative review articles can serve these purposes appropriately, previous work has questioned the appropriateness of the increasing number of graduate theses that include a systematic review as part of the academic output [4–7].

While learners are frequently prompted to pursue reviews by faculty members, sometimes those faculty do not have the skills or experience to mentor the students through the learning process. In such cases, students must learn the methods on their own and seek out the necessary guidance. Novice reviewers can learn how to plan out their review by reading about the methods in articles and handbooks, watching video tutorials, providing research assistance with a more experienced review team, participating in courses or workshops, or any combination of these strategies [8, 9].

In addition to formal learning opportunities and self-directed learning, students may receive guidance from methodological experts, including academic health sciences librarians. Novice reviewers frequently consult librarians for their search expertise [10]. Many librarians also provide support for other aspects of conducting and writing the review, including advice on refining the review question, instruction on the appropriate choice of review methodology, and guidance on data management issues [11, 12]. Wissinger commented on a perceived increase in contact between librarians and students participating on review teams, as well as the challenges involved when learners undertake their own systematic review projects [7].

There have been several recent reviews regarding both online and in-person SR training opportunities. These reviews summarize the in-person or blended training that has been reported in the literature up to 2020 [13] and the web-based courses, tutorials, and videos available in 2015 [9]. A wide variety of teaching interventions have been reported, such as instructional sessions with or without supplemental learning through web-based tutorials, homework, or follow-up [14, 15]. There have also been several published program descriptions or educational evaluations that report that academic librarians have been offering a range of SR searching instructional support for trainees [13–20].

Searching for evidence to include in SRs involves unique skills that correspond with, but do not exactly mirror, fundamental information literacy (IL) skills for general information retrieval nor evidence-based practice (EBP) skills of finding, evaluating, and integrating research evidence into clinical practice. There is extensive literature on the instruction efforts related to both former constructs, as demonstrated by systematic reviews on librarian-led IL and EBP instruction [21–23]. Examples also exist of research on the impact of library instruction on systematic searching skills [23] and academic research projects generally [24], showing positive correlations regardless of format or evaluation methods [25-26].

Individual case reports published across the literature provide some evidence of the impact of several models of library instruction on learner satisfaction and searching abilities, yet do not provide a broader depiction of librarian's teaching practices for comprehensive searching. For example, Premji et al.'s scoping review of knowledge synthesis instruction integrates librarians with a broader pool of KS instructors, while also excluding online education initiatives and didactics focused specifically on a single step (e.g. searching) of the review process [13]. Therefore, the cross-sectional summary of knowledge synthesis instruction as of 2021 gives an incomplete picture of librarian contributions to instruction in this domain. Meanwhile, there have been no investigations of the instructional practices of librarians across institutions in support of SRs and other comprehensive reviews, suggesting a gap in our understanding of teaching practices, content covered, and instructional formats of librarians when supporting trainees to search comprehensively.

With this study, we aimed to address this gap by surveying librarians to inventory the teaching practices used with groups of learners and answer the following research question: What are the teaching practices, content covered in instructional sessions, and resources used when academic health librarians teach groups of students comprehensive searching as needed for KS projects?

## METHODS

We conducted an exploratory-descriptive study using online survey data collection. The survey instrument can be found in Appendix A. A positionality statement outlining the researchers in relation to the context of the study can be found in Appendix B.

### Survey Development

An online survey was developed in SurveyMonkey. A first draft of the survey instrument was initially developed by two authors and then finalized by all authors. The survey questions were designed to collect non-identifying demographic data, to gather information regarding pedagogical approaches used when teaching, and to understand scope of content covered. The options for questions involving multiple choice selection were generated using a combination of author's subject expertise and targeted reviews of the literature. Recognizing that not all options could be pre-determined, each question included an “other” response option.

Ethics approval was obtained by the University of Toronto ethics review board in June 2022 (REB #43095). The survey was pilot tested by four individuals from different academic institutions, who were familiar with the subject matter and survey methodologies. The feedback from the pilot test was synthesized, and the survey items were modified accordingly. The survey instrument was finalized following the pilot test to include 31 open-ended and closed-ended (numerical range, categorical, and matrix scale) questions as described below. To avoid contributing to survey fatigue prior to collecting data related to our research questions, demographics questions unrelated to the inclusion criteria were asked at the end of the survey [28].

### Population

Branching logic was used to identify the respondents that met the elements of our population of interest which was health sciences librarians. Additional eligibility questions screened in respondents that 1) teach comprehensive searching for knowledge synthesis projects in 2) group settings.

The first two questions identified whether respondents met our base population. A librarian was defined as an individual who holds an MLIS, MI, or equivalent and was employed in a position where holding one of these degrees is required. Health sciences was defined as engaging with students in a degree program such as medicine, nursing, dentistry, public health, rehabilitation, kinesiology, pharmacy, or social work. Individuals not meeting these two elements were exited from the survey.

Next, participants were asked whether they teach comprehensive searching methods for knowledge synthesis projects. Comprehensive searching was defined as a reproducible and transparent search method that aims to identify every paper on a given research topic, accomplished through a search that is structured, operationalized, and executed using advanced features in a bibliographic database. Knowledge syntheses were defined as “the contextualization and integration of research findings of individual research studies within the larger body of knowledge on the topic” using reproducible and transparent methods [29]. Participants who selected ‘no’ or ‘unsure’ were asked to elaborate on why they were unsure and were then exited from the survey.

Finally, participants were asked whether they teach these topics in group settings. A group setting was defined as including 3 or more learners. Those who selected ‘no’ or ‘unsure’ were asked to explain why not or why they were unsure, then redirected to the demographic questions before exiting the survey.

The remaining participants represented our specific population of interest and were directed to answer the remaining 24 questions.

### Survey Distribution

The survey opened in August 2022 and was distributed electronically by email. The recruitment email can be found in Appendix C. The survey was distributed to a variety of librarian association electronic listservs, including: EAHIL, MEDLIBS, CANMEDLIBS, KSIG, AFMC, CILIP, aliaHEALTH, and MARIMEDLIB. The survey was also distributed on Twitter and Facebook. All questions in the survey were optional, and participants could choose to leave the survey at any time. The survey was open for one month, with a reminder email being sent halfway through the recruitment period, following Dillman's survey methodology for internet distribution [28].

### Analysis

Results from the survey were exported from SurveyMonkey into Excel for analysis. Closed-ended questions were analyzed using descriptive statistics. For the questions that had a narrative response component, a code book was established using thematic analysis [30]. To create the code book, one author scanned the data and developed draft codes based on two open-ended question responses. The draft codes were then reviewed and finalized by all authors. Following this, two authors independently coded the open-ended questions using the developed codes from the code book. The research team then met to discuss major ideas and codes generated from each question and across the open text responses.

#### Results

The survey instrument including exact questions asked can be found in Appendix A. For clarity as to which questions are being reported in each section of the results, we have indicated the question numbers throughout.

### Responses and Demographic Information (Questions 1, 2, 3, 4)

The survey received responses from a total of 114 participants, all of whom identified as a librarian. Of these, 105 respondents selected that they work with students in the health sciences. 90 respondents indicated that they teach comprehensive searching, but 16 of those did not report teaching the topic in a group setting. This left 74 respondents that met the target population of our survey, 57 of whom fully completed all questions. A schematic of this process can be found in Appendix D. The question response rate declined throughout the survey, so we have noted the response rate for each question throughout the results reported for the sake of clarity. We are not able to estimate the global number of academic health sciences librarians nor the number of recipients of the various means of distributing the invitation to participate, and therefore are unable to calculate a total response rate.

All respondents were asked to report on their length of career and country of employment. Respondents meeting all the inclusion criteria were also asked how long they had been teaching KS in group settings. These results are reported in Table 1.

**Table 1:** Responses to the length of career, country of employment, and years teaching KS in group settings (Questions 8, 21, 22).

Characteristic	All Respondents - no. (%)
Length of Career	n=69
1–5 years	9 (13)
6–10 years	14 (20)
11–15 years	12 (18)
Over 15 years	32 (46)
Country	n=68
Australia	2 (3)
Canada	33 (49)
Croatia	1 (1)
Ireland	2 (3)
Netherlands	2 (3)
New Zealand	1 (1)
Nigeria	1 (1)
Portugal	1 (1)
Spain	3 (4)
Sweden	4 (6)
Switzerland	1 (1)
United States	11 (16)
United Kingdom	6 (9)
Years Teaching KS	n=64
Less than 1 year	6 (9)
1–5 years	27 (42)
6–10 years	14 (22)
11–15 years	6 (9)
Over 15 years	11 (17)

### Barriers and Motivations (Questions 3A, 4A, 5)

From the 102 respondents who reported that they are health sciences librarians, 9 reported that they do not teach comprehensive searching as defined for this study and 3 responded they were unsure. In an open-ended question, they were asked why not, or why they were unsure. The most common responses were lack of support available at their institution, time limitations, and that this task did not fall within their job responsibilities. As they did not meet the inclusion criteria, these 12 individuals were then exited from the survey and no further data collected.

In an open-ended question, the remaining respondents were asked to provide one to three reasons why they teach comprehensive searching methods for KS in group settings. 151 responses from 63 respondents were coded using the coding dictionary. The most common codes selected were curriculum, student learning, and logistics. A summary of the frequency of codes, example responses, and code definitions can be found in Appendix E.

### Frequency and Group Size (Questions 6 and 7)

The majority (56%; n=36) of 64 respondents indicated they deliver a group workshop for knowledge synthesis searching 2 to 5 times a year. The total number of participants taught over the course of a typical year varied, with 17% (n=11) of librarians reporting 3 to 10 participants, 38% (n=24) reporting 11 to 50 participants, 25% (n=16) reporting 51 to 100 participants, and 17% (n=11) reporting 101 to 500 individuals. One respondent indicated they taught more than 500 individuals in a year.

### Locations and Format (Questions 9, 10, 13)

When asked about location, the majority of the 63 respondents teach online (87%, n=54), followed by in-person (79%, n=49) and hybrid (45%, n=28). An open text “other” response was also provided, where several respondents noted that the pandemic had impacted the locations where they teach, with more instruction occurring online than previously.

Respondents were also asked which formats they teach in. The majority of the 64 respondents (97%, n=57) teach completely synchronously. 46% (n = 29) teach using a mix of synchronous and asynchronous methods, and 17% (n=11) teach entirely asynchronously.

**Figure 1:**
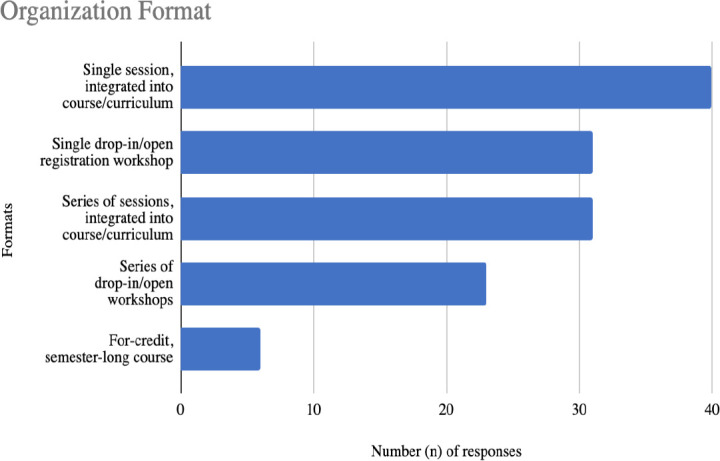
Quantitative responses to the question: What organization format do you use when teaching comprehensive searching methods for KS in group settings? Select all that apply.

Respondents were also asked how they organize the delivery of their instruction. The most common selection by the 62 respondents was a single session, integrated into a course or curriculum (67%, n = 40). Additional results can be seen in Figure 1. Most respondents indicated they taught in more than one type of format (66%, n = 41/62, range 1–5, average = 2.22).

### Tools and Activities (Questions 11, 12)

**Figure 2:**
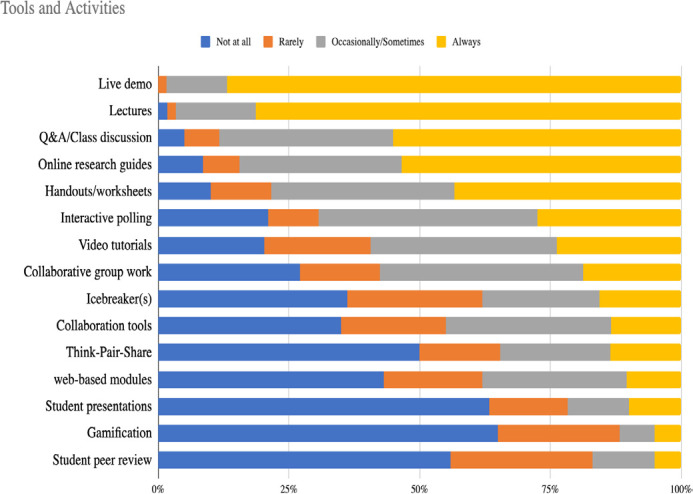
Responses to the question: In preparing for, delivering, or following up on group instructional sessions, how often do you use the following tools and activities to teach comprehensive searching methods for KS?

Respondents were asked to select which tools and activities they use to teach comprehensive searching in group settings. 15 options were provided, and respondents were asked to choose the frequency at which they used the tool or activity using the options of Not at all, Rarely, Occasionally/Sometimes, and Always. Live demonstrations were reported Always used by 87% (n=53) of the 62 respondents that answered, and this option had no responses for “Not at all”. More than 50% of responding librarians indicated they also always used lectures, class discussions, and online research guides. Additional results can be seen in Figure 2. 17 respondents gave other examples of teaching strategies, from storytelling to specific exercises or assignments, which are reported in Appendix F.

### Topics (Question 14)

A variety of questions were asked regarding the specific elements taught, as well as how those elements are integrated into teaching. Respondents were given a list of 32 topics and asked whether they include it in their sessions. All 32 topics were selected at least once. The most common topics covered were Boolean operators (100%, n = 56), database selection, synonym generation, controlled vocabulary, and executing a database search (98%, n = 55). Appendix G illustrates additional results regarding the frequency of respondents who cover each of the topics, sorted in order of most frequent to least.

For each topic, 56 respondents were also asked how they integrate the topic into their teaching to gather impressions of which topics were covered by a range of didactic, self-directed, and active learning strategies. Of note, four of the five search topics noted above are also the topics with the most dynamic teaching approaches reported. Full results are presented in Table 2.

**Table 2:** Illustrates how different topics are included when teaching comprehensive search methods for KS in group settings. Results are presented on a colour spectrum with red being the least frequent, yellow being the median, and blue being the most frequently selected. The results are sorted with the most frequently selected topics at the top.

	Method and frequency (n) of inclusion				
Topics	I Define the Topic	I provide a reading	I provide how-to guidance	I conduct a demonstration	I use active learning
Boolean logic (n=56)	33	14	41	49	32
Executing a database search (n=55)	23	17	39	49	31
Controlled vocabulary eg. MeSH, Emtree (n=55)	32	17	42	49	30
Synonym generation (n=55)	27	16	40	43	33
Database selection (n=55)	29	24	35	31	19
Translating search strategies (n=54)	31	25	27	30	16
Database syntax (n=53)	29	20	35	41	27
Search documentation (n=53)	35	36	34	23	12
Question formulas eg. PICOTT, PCC, SPIDER (n=52)	25	22	23	31	24
Refining review question (n=52)	30	19	24	26	21
Determining appropriate review type (n=52)	25	35	20	12	8
Reporting guidelines eg. PRISMA (n=52)	24	38	23	10	6
Conduct/methodological guidance eg. Cochrane MECIR standards, JBI Manual (n=52)	23	42	17	9	4
Testing search terms (n=51)	26	13	34	39	24
Search filters (n=51)	36	28	23	30	11
Citation management software eg. Endnote, RefWorks (n=51)	31	35	25	22	15
Deduplication (n=51)	37	24	19	19	9
Grey literature (n=51)	37	34	16	16	5
Clinical trial registries (n=49)	36	31	14	12	5
Screening (n=47)	32	27	18	13	7
Sensitivity vs. precision (n=46)	28	13	17	22	10
Protocol creation (n=45)	29	36	16	8	4
Evidence-based medicine (n=45)	32	20	12	7	5
Systematic review management software eg. DistillerSR, Covidence (n=44)	29	31	15	16	4

### Pedagogy (Questions 15, 16, 17, 19)

A variety of questions were asked related to education pedagogy and teaching approaches. Respondents were asked which educational frameworks they use when developing or refining teaching. Nine options were provided in addition to an “other” text response, with respondents selecting all that applied. 61% (n= 34) of the 55 respondents answered that they do not use any specific resources or frameworks. For those that do use a pedagogical model, the ACRL Framework for Information Literacy for Higher Education was the most frequent selection (27%, n =15). Textual responses in the “other” category included additional frameworks, specifically the SCONUL framework, adult learning theory, SOLO taxonomy, and Kolb's learning cycle.

**Figure 3:**
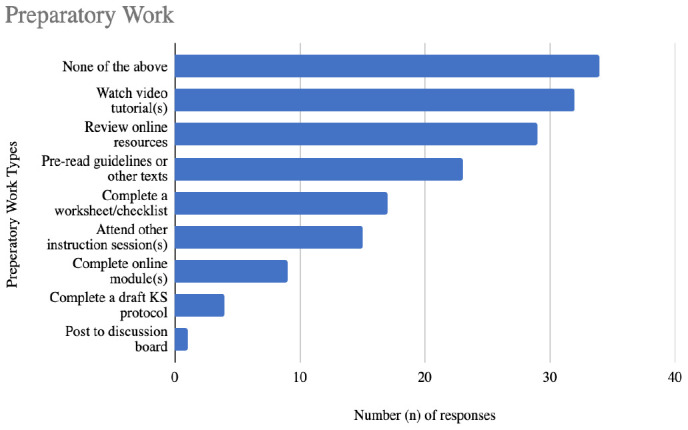
Quantitative responses to the question: When teaching comprehensive searching methods for KS in group settings, what, if any, preparatory work do you typically assign for learners to complete prior to the instructional encounter? Select all that apply.

Respondents were also asked what preparatory work they typically assign to learners to complete in advance of the instructional encounter. Nine options were provided in addition to an “other” response. The majority of the 56 respondents (61%, n = 34) did not use any of the preparatory work options that were listed. The open text “other” response indicated that preparatory work was sometimes provided as an optional, but not mandatory activity. Asking students to come prepared with their own developed research question was also mentioned. Additional results are reported in Figure 3.

Respondents were asked whether they state learning objectives or learning outcomes, either provided orally or presented visually on slides. Most do, with 66% (n = 37) of the 56 respondents selecting always, 23% (n =13) responding sometimes, 7% (n = 4) responding rarely, and 3% (n =2) responding never.

For what types of support librarians provide to learners following the educational encounter, six options were provided to respondents to select from, as well as an “other” textual response option. Two follow-up supports were selected the most frequently by the 57 respondents: one-on-one consultations (89%, n= 51) and online resources such as library research guides or websites (89%, n = 51). All the other support options were also frequently selected, specifically contact information (87%, n = 50), lecture slides (84%, n = 48), and video tutorials (61%, n = 35). All the respondents selected at least one of the additional support options provided.

### Assessment (Question 18, 20)

**Figure 4:**
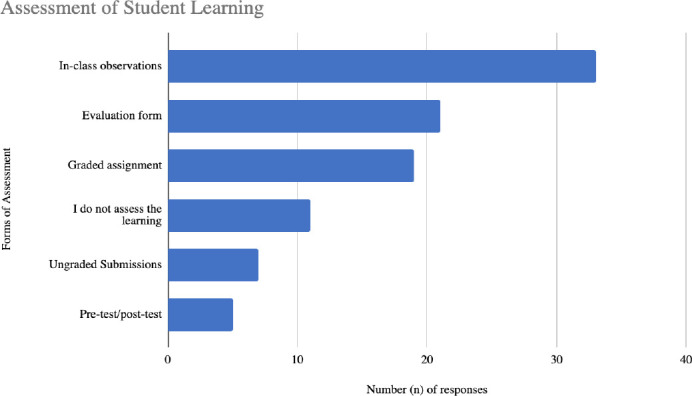
Quantitative responses to the question: When teaching comprehensive searching methods for KS in group settings, how do you assess student learning? Select all that apply.

Respondents were asked how they assess student learning. Six options were provided in addition to an “other” write-in option and they were asked to select all that apply. The most common response, with 60% (n = 33) of the 55 respondents, was in-class observations (such as class participation and informal feedback). Evaluation forms such as a ticket out the door or exit survey were the second most common form of assessment with 38% (n=21) of the 55 respondents selecting this option. Additional results are reported in Figure 4.

**Figure 5:**
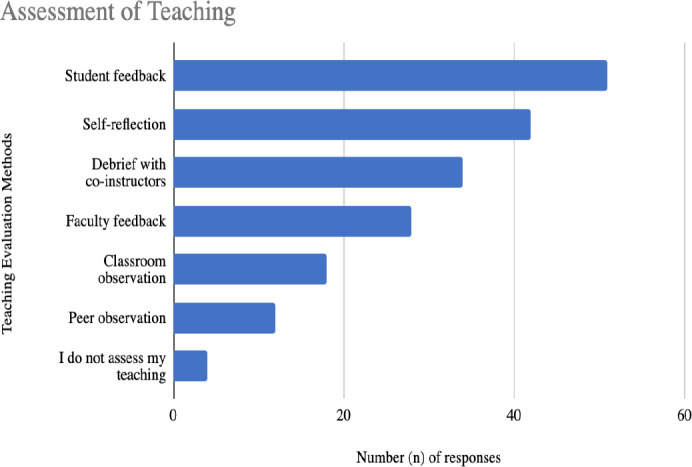
Quantitative responses to the question: How do you assess your teaching of comprehensive searching methods for KS in group settings? Select all that apply.

Respondents were also asked how they assess their own effectiveness as teachers. Seven options were provided in addition to an “other” option, with the prompt to select all that applied. Most respondents 92% (n = 53/57) indicated that they assess their teaching in some way, most frequently using student feedback (89%, n = 51) and self-reflection (73%, n =42) as methods to do so. Additional results are reported in Figure 5.

## DISCUSSION

The findings from this study add to our understanding of the teaching practices, content covered in instructional sessions, and resources used when academic health librarians teach groups of students to comprehensively search for KS projects. We have learned from survey respondents that some factors can act as either motivations or barriers, such as available time, the individual's job description, and demand from learners or instructors. The responses have highlighted trends in the content, teaching approaches, and contexts of the instruction provided by librarians.

By using a cross-sectional approach to inventory instructional practices across institutions and countries, we have added to what is known about common practices and revealed trends in KS searching instruction. Similar to other recent explorations of research support and instruction in health sciences libraries, our findings reflect current practices, including online instruction and multiple, concurrent strategies for supporting learners [32]. Other than the collation of separate descriptions in the scoping review published by Premji et al., this study is the first to look cross-institutionally at the teaching practices of librarians involved in KS instruction [13]. Furthermore, since the selection criteria of the review excluded both single-topic (such as searching) and online instruction, our study captures a broader range of instruction reflective of librarian teaching practices [13].

Aligning with other program descriptions [15-19] and Premji et al.'s review [13], librarians in this study reported focusing on search-related skills, such as identification of appropriate databases, text and index terms, and constructing the search syntax; however, other elements of KS methods were also frequently included. Whereas the librarian-led studies included in the review covered mainly the search and question defining steps [13, 15, 16], our study found librarians report including important non-search aspects of KS projects, such as the overall methods, reporting guidance, developing a protocol, and selecting an appropriate review methodology. However, compared with searching skills, these other concepts were more often referenced by providing a reading or definition rather than by demonstration or active learning strategies.

Notably, our results suggest that librarian's application of formal pedagogical approaches while teaching KS methodologies may be under-utilized. A minority of respondents in this study reported integrating well-known educational frameworks in the design of their instruction. Similarly, few respondents used standardized assessment of learner outcomes, assignment of pre-work, or active learning. These findings should be interpreted in the context of the limited time and pedagogical strategies common in the one-shot style of instruction that predominated the survey responses. Recent reviews of teaching in academic libraries have identified similar gaps regarding the integration of instructional design principles and models, suggesting this shortcoming is not limited to teaching comprehensive searching or KS methods [33, 34]. Librarians providing KS methods and comprehensive searching instruction to groups should increase their use of instructional design principles noted in this research and modelled in published reports of search instruction [15-19, 30]. For example, one recent case presentation of a credit course developed by librarians used self-determination theory to frame their assessment and delivery [16] and another report highlighted scaffolding as a key element of their workshop series design [31]. Integration of instructional frameworks in workshops and one-shot sessions can guide decisions regarding the design and delivery of the instruction and increase confidence in the effectiveness of the teaching and impact on learner outcomes.

The findings of this survey illustrate that some types of instruction, such as the series of open registration or drop-in workshops reported by Hayden et al. [31], Fuller et al. [17], and Lenton and Fuller [16], and for-credit, full course offerings [18] may be less common than the single session seminars or webinars reported by other librarians [19, 35]. Furthermore, while these program descriptions emphasize active learning and scaffolded exercises, our findings suggest that much of the teaching on advanced searching techniques and KS methods relies on more passive means of delivering content, such as lectures and demonstrations. These two observations are likely linked, along with the prevalence of online instruction reported in our study, in that librarians may feel they have limited time in one-shot instruction and similarly have constraints on the teaching approaches to engage learners in online settings [36].

Of the seventeen studies summarized by Premji and colleagues, all educational interventions reported teaching searching and 14 of those included hands-on activities or experiential learning [13]. Likewise, the searching components reported in our study were the topics most likely to be taught through activities and exercises as well as other modes of delivery, suggesting librarians prioritized their engagement efforts around searching competencies. However, even the concepts and skills related to the search were not always taught through learner-centered pedagogies, including important elements such as documenting the search. Instructional program descriptions highlighting active learning techniques for comprehensive searching may not be reflected in everyday teaching practices, particularly for stand-alone workshops or one-shot sessions [17-19, 31].

Although a recent scoping review shows that very few studies of educational interventions have reported the impact of group or individual instruction on literature searching skills [14], over a third of the librarians in this sample used at least some type of student evaluation form and only 20% do not assess learner outcomes in any way. Nonetheless, informal and subjective observation of student activities and behaviors in class were the most reported means of assessing learner response, followed by students' self-reported satisfaction; such approaches provide less reliable evidence of impact compared to objectively measured changes in behaviours or knowledge. While limited student assessment options are understandable given the constrained amount of time to engage and the nature of the guest lecture or one-shot session, the usefulness of such methods is further compromised when teaching online, where student engagement can be harder to elicit and observe. This study was not designed to evaluate the effectiveness of teaching practices, but we noted that the reliance on informal observations for learner assessment limits the ability of librarian teachers to determine the impact of their instructional sessions.

This study adds considerable detail to what we know about the content and approaches in group instruction sessions on KS methods taught by librarians. Reported instruction emphasized search skills, predominantly using demonstration, lecture, and - to a lesser degree - active learning, aligning with the findings of other research related to health librarian instruction for information literacy and evidence-based practice [21,22]. However, five of the 18 topics covered by at least 90% of respondents pertained to other aspects of the review process (i.e., refining the review question, frameworks for question formulation, determining appropriate review methodology, and reporting and conduct guidance), reflecting an appreciation of the interconnected steps when conducting KS research. Similarly, while librarian-led demonstrations and lectures were the most frequent forms of teaching, our results demonstrate that referring learners to self-directed learning tools, such as library research guides and video tutorials were also common strategies. The use of these resources as pre-work echoes the flipped classroom approaches to teach these topics, as reported by others [17, 19]. Likewise, engaging active learning approaches such as the use of worksheets, polls, and collaborative group work were reported to be employed at least sometimes by most respondents, correlating with other reported KS methods instruction approaches from the studies included in the review by Premji et al. [13].

### Limitations

While this study includes responses from multiple librarians at numerous institutions, it is only representative of those librarians who completed the survey. The length of the questionnaire may have deterred some potential respondents, as at least one person noted survey fatigue by the end of the form. There was also a decline in the response rate as the survey continued. Therefore, our findings may only be representative of some contexts and perspectives and may not be generalizable.

We recognize we sacrificed richness of data for breadth of reach as this type of survey also relies on self-report and individual recall, rather than observation or in-depth description of specific teaching experiences. Furthermore, in addressing our research questions related to group instruction practices, we explicitly excluded one-to-one research consultations. Knowledge synthesis instruction through individual consultations has been acknowledged as a significant means of supporting students and other researchers. This was noted both by the respondents of this survey, as well as in the authors' experiences, and aligns with what has been noted in the literature [20].

Similarly, this study does not examine the impact of advanced searching instruction on learner outcomes or outputs, an area of interest that currently lacks evidence, as noted in the recent scoping review of literature search instruction [14]. It is also possible that some survey responses were affected by the recent pandemic, as respondents to our survey noted the same increase in online instruction that was reported across health library teaching, but this relationship was not explored in our data [32].

### Future Research

The findings of this survey suggest possible directions for further research regarding the tools, approaches, and content employed by health librarians when providing support to individual learners, either through a similar survey or by ethnographic observations of librarians during research consultations. There are no existing measures of KS competencies for health professional trainees, though there has been work done to determine the competencies needed by librarians who support these projects [37] and descriptions and evaluations of the training for librarians related to SRs [38–40]. Likewise, some research has been done to develop measures of search expertise [41–44], but these measures have not been applied in the population of health professions trainees in the context of conducting KS projects. For example, unlike evidence-based practice competencies that focus on individual abilities [45], KS methods guidance emphasizes research teams with collective expertise, so developing advanced searching skills is less important than building an understanding of what thorough and systematic search strategies entail. The results from this survey will also allow for later exploration on the effectiveness of specific training interventions regarding the search skills, research outputs, or other research competencies of students working on KS projects. Understanding the effectiveness and role of librarian-led training will allow a better demonstration of the value health sciences librarians are bringing not only to the KS research landscape, but to the educational experiences of health science students as well.

### Conclusions and Implications for Practice

The educational support provided by librarians is important for students who are encouraged to conduct comprehensive reviews so they may become competent researchers and critical and thoughtful consumers of reviews. These findings may inspire librarians to expand their instruction beyond single sessions for individual courses or programs, to devote more time to active learning, and incorporate more structured approaches to designing sessions, assessing learner outcomes, and evaluating impact. Prioritizing time and effort with learners to build the technical skills and conceptual knowledge related to comprehensive search strategy development specifically utilizes librarian expertise. Meanwhile, instruction that links content related to other steps of review methods and research processes generally helps scaffold and contextualize learning about KS methods. Collaborating with supervisors, faculty, and other synthesis methodologists can help librarians coordinate instruction for groups of health sciences learners to align objectives, assess learner needs, and extend their impact on student success in the context of KS research. In combination with published program descriptions, our research provides librarians with examples of teaching strategies and content from which to select when designing or expanding instruction related to comprehensive searching and KS methods.

## Data Availability

Data associated with this article are available in the Open Science Framework at https://osf.io/7h3pt/.

## SUPPLEMENTAL FILES

**Appendix A:** Complete Survey

**Appendix B:** Positionality Statement

**Appendix C:** Recruitment Email

**Appendix D:** Methodology Schematic

**Appendix E:** Table Illustrating Frequency of Codes

**Appendix F:** Responses for “Other” Tools Used

**Appendix G:** Table Illustrating the Frequency of Respondents Who Cover Different Topics, Sorted in Order of Most Frequent to Least
