# Supplementary material for: Exploring librarians' practices when teaching advanced searching for knowledge synthesis: results from an online survey
Source: J Med Libr Assoc. 2024 Jul 29;112(3):238–49. doi: 10.5195/jmla.2024.1870 (PMC11412128; doi:10.5195/jmla.2024.1870)
Supplement: Supplementary file 1 — Appendix A: Complete Survey [file jmla-112-3-238-s01.pdf]

Librarian Group Instruction Practices for Knowledge Syntheses

CONSENT FORM

You are being invited to volunteer in a research study.

Our goal is to identify the approaches and resources health science librarians employ when teaching students how to conduct comprehensive searches for knowledge synthesis projects (eg. Systematic or scoping reviews) in group settings.

As a health science librarian, or librarian who works with health science students/trainees, you are being invited to participate and share your approach and experience teaching students how to search for knowledge syntheses in group settings.

Survey participants will complete an approximately 20-minute survey asking about their instructional practices and experiences supporting knowledge synthesis research by health science students/trainees.

The survey will remain open until September 19, 2022.

The confidentiality of your electronic data created by you or the researchers will be maintained by applicable law and to the degree permitted by the technology used. Absolute confidentiality cannot be guaranteed.

The survey questions will not be asking you for any personally identifiable information.

Information collected in this survey may be made openly available once the study is complete in the form of a publication, but we will not share any information in a way that could be combined to identify you.

Study authors received approval through the University of Toronto Social Sciences, Humanities, and Education Research Ethics Board. If you have questions, complaints, or concerns about the research, you may email the survey authors, erica.lenton@utoronto.ca and glyneva.bradley.ridout@utoronto.ca, Gerstein Science Information Centre, University of Toronto Libraries.

Your participation is voluntary and you may decide to stop at any time. If you have questions about your rights as participants, please contact the Research Oversight and Compliance Office – Human Research Ethics Program at ethics.review@utoronto.ca or 416-946-3273.

By beginning the survey, you are implying your voluntary consent to participate in

the research.

Librarian Group Instruction Practices for Knowledge Syntheses

1. Are you a librarian?

For the purposes of this survey, “librarian” is defined as an individual who holds an MLIS, MI, or equivalent, AND currently is employed in a position where holding an MLIS, MI, or equivalent is required.

- ◯ Yes
- ◯ No
- ◯ Unsure

2. Do you work with students enrolled in one or more health sciences degree program(s)?

For the purposes of this survey, we are considering the following health science degree programs: dentistry, kinesiology, medicine, nursing, pharmacy, psychology, public health, rehabilitation, and social work.

- ◯ Yes
- ◯ No
- ◯ Unsure

Librarian Group Instruction Practices for Knowledge Syntheses

3. As part of your librarian role, have you taught comprehensive search skills for the purposes of conducting a knowledge synthesis (KS) review?

For the purposes of this survey, “comprehensive searching” is defined as a reproducible and transparent search method that aims to identify every paper on a given research topic. This is accomplished through a search that is structured, operationalized, and executed using advanced features in a database.

“Knowledge synthesis” is defined as the contextualization and integration of research findings of individual research studies within the larger body of knowledge on the topic. A synthesis must be reproducible and transparent in its methods, using quantitative and/or qualitative methods (CIHR definition). Systematic reviews, scoping reviews, rapid reviews, and realist reviews are examples of KS projects.

- ◯ Yes
- ◯ No
- ◯ Unsure

3A. Please explain why not, or why you are unsure.

Librarian Group Instruction Practices for Knowledge Syntheses

4. Do you teach, or have you taught, comprehensive searching methods for KS in group settings (3 or more learners)?

- ◯ Yes
- ◯ No
- ◯ Unsure

4A. Please explain why not, or why you are unsure.

Librarian Group Instruction Practices for Knowledge Syntheses

5. What are 1-3 reasons why you teach comprehensive searching methods for KS in group settings?

Reason 1

Reason 2

Reason 3

6. How many times per year, on average, do you deliver a workshop/series on search methods for KS in group settings?

- Less than once a year
- Once a year
- 2-5 times a year
- 5 or more times a year

7. In a typical year, how many learners combined do you estimate you teach search methods for KS in group settings (not counting individual consultations)?

- 3-10
- 11-50
- 51-100
- 101-500
- more than 500

8. In your role as a librarian, how long have you been teaching comprehensive searching methods for KS in group settings?

- Less than 1 year
- 1-5 years
- 6-10 years
- 11-15 years
- more than 15 years

Librarian Group Instruction Practices for Knowledge Syntheses

9. When teaching group sessions on comprehensive searching methods for KS, in which location(s) have you taught?

Select all that apply:

- [ ] Online
- [ ] In person
- [ ] Hybrid online and in-person

Other, describe:

10. When teaching group sessions on comprehensive searching methods for KS, in which format(s) have you taught?

Select all that apply:

- [ ] Completely synchronous (eg. Live lecture)
- [ ] Completely asynchronous (eg. Self-paced module)
- [ ] Mix of synchronous and asynchronous (eg. Flipped classroom)

Other, describe:

11. In preparing for, delivering, or following up on group instructional sessions, how often do you use the following tools and activities to teach comprehensive searching methods for KS?

	Not at all	Rarely	Occasionally/Sometimes	Always	Unsure
Video tutorials	◯	◯	◯	◯	◯
Interactive web-based modules	◯	◯	◯	◯	◯
Handouts/worksheets	◯	◯	◯	◯	◯
Lectures	◯	◯	◯	◯	◯
Live demo	◯	◯	◯	◯	◯
Online research guides (eg. LibGuides)	◯	◯	◯	◯	◯
Collaboration tools (eg. Padlet, Miro, Google Suite, whiteboards, chart paper)	◯	◯	◯	◯	◯
Interactive polling (eg. Raised hands, polling software, Zoom polls)	◯	◯	◯	◯	◯
Gamification (eg. Competitions, Kahoot, Scavenger hunt, rewards)	◯	◯	◯	◯	◯
Collaborative group work	◯	◯	◯	◯	◯
Student presentations	◯	◯	◯	◯	◯
Student peer review	◯	◯	◯	◯	◯
Question and answer/Class discussion	◯	◯	◯	◯	◯
Think-Pair-Share	◯	◯	◯	◯	◯
Icebreaker(s)	◯	◯	◯	◯	◯

12. Do you use any other instructional tools/activities to teach comprehensive searching methods for KS in group settings? If yes, please describe.

◯ No

◯ Yes (please describe)

13. What format do you use when teaching comprehensive searching methods for KS in group settings?

Select all that apply:

- ☐ Single drop-in/open registration workshop
- ☐ Series of drop-in/open registration workshops
- ☐ Single session, integrated into course/program curriculum
- ☐ Series of sessions, integrated into course/program curriculum
- ☐ For-credit, semester-long course

Other, describe:

Librarian Group Instruction Practices for Knowledge Syntheses

14A. Review the list of topics below. For each, check the boxes to indicate how you typically include that topic when teaching comprehensive search methods for KS in group settings.

Select all that apply for each topic:

	I do not include this topic in my session(s)	I define the topic	I provide further readings/supplementary materials for this topic	I provide step-by-step how-to guidance on the topic	I demonstrate the topic in a live demonstration	I facilitate an activity where students can practice/discuss the topic	I am unsure how I include this topic in my session(s)
Hierarchy of evidence	☐	☐	☐	☐	☐	☐	☐
Evidence-based medicine	☐	☐	☐	☐	☐	☐	☐
Determining appropriate review type	☐	☐	☐	☐	☐	☐	☐
Reporting guidelines (eg. PRISMA)	☐	☐	☐	☐	☐	☐	☐
Conduct/methodological guidance (eg. Cochrane MECIR standards, JBI Manual)	☐	☐	☐	☐	☐	☐	☐

14B. Review the list of topics below. For each, check the boxes to indicate how you typically include that topic when teaching comprehensive search methods for KS in group settings.

Select all that apply for each topic:

	I do not include this topic in my session(s)	I define the topic	I provide further readings/supplementary materials for this topic	I provide step-by-step how-to guidance on the topic	I demonstrate the topic in a live demonstration	I facilitate an activity where students can practice/discuss the topic	I am unsure how I include this topic in my session(s)
Protocol creation	☐	☐	☐	☐	☐	☐	☐
Refining review question	☐	☐	☐	☐	☐	☐	☐
Question formulas (eg. PICOTT, PCC, SPIDER)	☐	☐	☐	☐	☐	☐	☐
Sensitivity vs. precision	☐	☐	☐	☐	☐	☐	☐
Database selection	☐	☐	☐	☐	☐	☐	☐

14C. Review the list of topics below. For each, check the boxes to indicate how you typically include that topic when teaching comprehensive search methods for KS in group settings.

Select all that apply for each topic:

	I do not include this topic in my session(s)	I define the topic	I provide further readings/supplementary materials for this topic	I provide step-by-step how-to guidance on the topic	I demonstrate the topic in a live demonstration	I facilitate an activity where students can practice/discuss the topic	I am unsure how I include this topic in my session(s)
Synonym generation	☐	☐	☐	☐	☐	☐	☐
Controlled vocabulary (eg. MeSH, Emtree)	☐	☐	☐	☐	☐	☐	☐
Boolean logic	☐	☐	☐	☐	☐	☐	☐
Database syntax	☐	☐	☐	☐	☐	☐	☐
Testing search terms	☐	☐	☐	☐	☐	☐	☐

14D. Review the list of topics below. For each, check the boxes to indicate how you typically include that topic when teaching comprehensive search methods for KS in group settings.

Select all that apply for each topic:

	I do not include this topic in my session(s)	I define the topic	I provide further readings/supplementary materials for this topic	I provide step-by-step how-to guidance on the topic	I demonstrate the topic in a live demonstration	I facilitate an activity where students can practice/discuss the topic	I am unsure how I include this topic in my session(s)
Test sets	☐	☐	☐	☐	☐	☐	☐
Translating search strategies	☐	☐	☐	☐	☐	☐	☐
Executing a database search	☐	☐	☐	☐	☐	☐	☐
Grey literature	☐	☐	☐	☐	☐	☐	☐
Clinical trial registries	☐	☐	☐	☐	☐	☐	☐

14E. Review the list of topics below. For each, check the boxes to indicate how you typically include that topic when teaching comprehensive search methods for KS in group settings.

Select all that apply for each topic:

	I do not include this topic in my session(s)	I define the topic	I provide further readings/supplementary materials for this topic	I provide step-by-step how-to guidance on the topic	I demonstrate the topic in a live demonstration	I facilitate an activity where students can practice/discuss the topic	I am unsure how I include this topic in my session(s)
Reporting bias	☐	☐	☐	☐	☐	☐	☐
Citation management software (eg. Endnote, RefWorks)	☐	☐	☐	☐	☐	☐	☐
Systematic review management software (eg. DistillerSR, Covidence)	☐	☐	☐	☐	☐	☐	☐
Deduplication	☐	☐	☐	☐	☐	☐	☐
Search documentation	☐	☐	☐	☐	☐	☐	☐

14F. Review the list of topics below. For each, check the boxes to indicate how you typically include that topic when teaching comprehensive search methods for KS in group settings.

Select all that apply for each topic:

	I do not include this topic in my session(s)	I define the topic	I provide further readings/supplementary materials for this topic	I provide step-by-step how-to guidance on the topic	I demonstrate the topic in a live demonstration	I facilitate an activity where students can practice/discuss the topic	I am unsure how I include this topic in my session(s)
Search filters	☐	☐	☐	☐	☐	☐	☐
Screening	☐	☐	☐	☐	☐	☐	☐
Data extraction	☐	☐	☐	☐	☐	☐	☐
Critical appraisal	☐	☐	☐	☐	☐	☐	☐
Quantitative analysis/meta-analysis of results	☐	☐	☐	☐	☐	☐	☐

14G. Review the list of topics below. For each, check the boxes to indicate how you typically include that topic when teaching comprehensive search methods for KS in group settings.

Select all that apply for each topic:

	I do not include this topic in my session(s)	I define the topic	I provide further readings/supplementary materials for this topic	I provide step-by-step how-to guidance on the topic	I demonstrate the topic in a live demonstration	I facilitate an activity where students can practice/discuss the topic	I am unsure how I include this topic in my session(s)
Qualitative analysis of results	☐	☐	☐	☐	☐	☐	☐
Journal submission requirements	☐	☐	☐	☐	☐	☐	☐

Other (please specify)

[ ]

15. What resources/frameworks do you use when developing/refining your teaching of comprehensive searching methods for KS in group settings?

Select all that apply:

- [ ] ACRL Framework for Information Literacy for Higher Education
- [ ] ADDIE Model
- [ ] Backward Design
- [ ] Bloom's Taxonomy
- [ ] Gagne's Nine Events of Instruction
- [ ] Merrill's Principles of Instruction
- [ ] Teaching Tripod Approach
- [ ] Universal Design for Learning
- [ ] I did not use a specific resource or framework

Other, describe:

Librarian Group Instruction Practices for Knowledge Syntheses

16. When teaching comprehensive searching methods for KS in group settings, what, if any, preparatory work do you typically assign for learners to complete prior to the instructional encounter?

Select all that apply:

- ☐ Attend other instruction session(s)
- ☐ Watch video tutorial(s)
- ☐ Complete online module(s)
- ☐ Complete a worksheet(s)/checklist
- ☐ Review online resources—eg. online research guides (eg. LibGuides), FAQs, websites
- ☐ Pre-read methods guidelines or other texts
- ☐ Post to discussion board
- ☐ Complete a draft KS protocol
- ☐ None of the above

Other, describe:

17. When teaching comprehensive searching for KS in group settings, do you state learning objectives/outcomes (eg. provided orally and/or presented on your slides?)

- ○ Not at all
- ○ Rarely
- ○ Occasionally/sometimes
- ○ Always

18. When teaching comprehensive searching methods for KS in group settings, how do you assess student learning?

Select all that apply:

- ☐ Evaluation form (eg. Ticket-out-the-door, exit survey)
- ☐ In-class observations (eg. Class participation, informal feedback)
- ☐ Student performance on related graded assignment
- ☐ Quality of student submissions (ungraded)
- ☐ Pre-test/post-test
- ☐ I do not assess the learning

Other, describe:

19. When teaching comprehensive searching methods for KS in group settings, what follow-up supports are available to learners?

Select all that apply:

- ☐ One-on-one consultation(s)
- ☐ Video tutorials
- ☐ Lecture slides
- ☐ Online resources—eg. online research guides (eg. LibGuides), FAQs, websites
- ☐ Contact information—eg. email address, phone number
- ☐ None of the above

Other, describe:

20. How do you assess your teaching of comprehensive searching methods for KS in group settings?

Select all that apply:

- [ ] Self-reflection
- [ ] Debrief with co-instructors
- [ ] Student feedback
- [ ] Faculty feedback
- [ ] Peer-observation
- [ ] Classroom observation
- [ ] I do not assess my teaching of comprehensive searching methods for KS

Other, describe:

Librarian Group Instruction Practices for Knowledge Syntheses

21. How long have you been a librarian?

- Less than 1 year
- 1-5 years
- 6-10 years
- 11-15 years
- more than 15 years

22. What country do you currently work in?

23. Do you have any other thoughts you would like to share related to teaching comprehensive searching methods for KS in group settings?

Thank you for completing the survey!
