# Supplementary material for: Exploring librarians' practices when teaching advanced searching for knowledge synthesis: results from an online survey
Source: J Med Libr Assoc. 2024 Jul 29;112(3):238–49. doi: 10.5195/jmla.2024.1870 (PMC11412128; doi:10.5195/jmla.2024.1870)
Supplement: Supplementary file 2 — Appendix B: Positionality Statement [file jmla-112-3-238-s02.docx]

**Positionality Statement**

Positionality is the positioning of the researcher in relation to the context of the study. The position adopted by a researcher affects every phase of the research process, from the way the question is initially constructed, to how others are invited to participate, how the research is conducted and, finally, how outcomes are disseminated and published [27]. In the case of this project, most of the authors are academic health science librarians who teach KS methods on a regular basis and are motivated to understand how their own teaching practices fit with those of other librarians and at other institutions. Two authors are also PhD candidates working on doctoral research related to the teaching and learning of KS methods; their research uses qualitative and mixed methods designs. One author is a Master of Information candidate currently studying library and information sciences with experience as an intern at an academic health sciences library.
