# Supplementary material for: Exploring librarians' practices when teaching advanced searching for knowledge synthesis: results from an online survey
Source: J Med Libr Assoc. 2024 Jul 29;112(3):238–49. doi: 10.5195/jmla.2024.1870 (PMC11412128; doi:10.5195/jmla.2024.1870)
Supplement: Supplementary file 3 — Appendix C: Recruitment Email [file jmla-112-3-238-s03.docx]

## Appendix C: Recruitment Email

Dear colleague,

You are invited to participate in a survey on health sciences librarians’ teaching practices in support of student knowledge syntheses projects.

The goal of the project is to identify a) whether health science librarians teach students knowledge synthesis search methods in group settings and b) if they do, what approaches and resources they employ when teaching.

You qualify to participate in this study if:

- You are a librarian currently employed in a health sciences library and/or you work directly with health sciences students and faculty
- For the purposes of this survey, we are considering the following health science degree programs: dentistry, kinesiology, medicine, nursing, rehabilitation, pharmacy, psychology, public health, and social work.

The results of this research will be of interest to library and information professionals who want to know more about the approaches and educational resources that health librarians use in group settings to help health professional trainees develop comprehensive searching skills for conducting knowledge synthesis projects. This research will contribute to presentations and publications within the Library and Information Science community. There are no anticipated harms to participating in the study.

The survey will not collect any personally identifiable information. By completing the survey, you are giving your consent to participate. This survey will take approximately 20 minutes to complete.

Should you choose to participate, please proceed to the survey by clicking on this link: [link to survey]

This survey closes on September 19, 2022.

This research has been approved by the University of Toronto Ethics Board. Please contact the research team if you have any questions.

Thank you.
