# Supplementary figures and images for: Exploring librarians' practices when teaching advanced searching for knowledge synthesis: results from an online survey
Source: J Med Libr Assoc. 2024 Jul 29;112(3):238–49. doi: 10.5195/jmla.2024.1870 (PMC11412128; doi:10.5195/jmla.2024.1870)

## Appendix D: Methodology Schematic


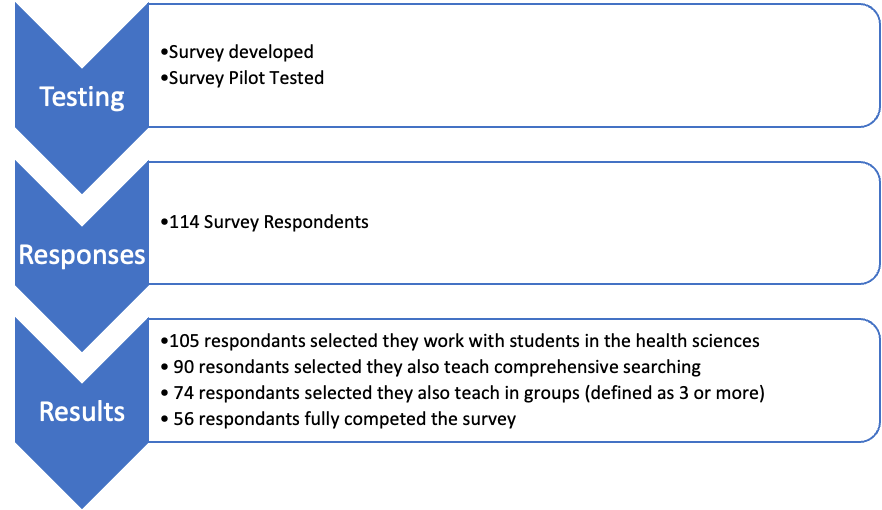

Supplement: Supplementary file 4 — Appendix D: Methodology Schematic [file jmla-112-3-238-s04.docx]
