# Supplementary material for: Exploring librarians' practices when teaching advanced searching for knowledge synthesis: results from an online survey
Source: J Med Libr Assoc. 2024 Jul 29;112(3):238–49. doi: 10.5195/jmla.2024.1870 (PMC11412128; doi:10.5195/jmla.2024.1870)
Supplement: Supplementary file 5 — Appendix E: Table Illustrating Frequency of Codes [file jmla-112-3-238-s05.docx]

## Appendix E: Table illustrating frequency of codes to the question “Provide 1-3 reasons why you teach comprehensive searching methods for KS in group settings”

| Codes | Definition from code book | Frequency (n) | Selected Examples |
| --- | --- | --- | --- |
| Curriculum | Teaching is (or is not) required as part of a course, curriculum, program, thesis, or research project | 47 | “It is one of the points in the education process where learners are thoroughly engaged in serious searching, so it is an opportunity to make sure that they have that knowledge” |
|  |  |  | “I teach comprehensive searching methods for KS to students… that are enrolled in a course that initiates them to the systematic review process, which includes searching for studies” |
| Student Learning | Teaching does (or does not) influence the student learning experience | 44 | “Students learn from each other’s research questions” |
|  |  |  | “It is essential to researchers being able to either do the search or know how to properly define a search for someone else to do it” |
|  |  |  | “Group reflection enhances learning” |
| Logistics | Teaching is (or is not) done because of scheduling, logistics, effort, or cost | 40 | “Overload of consults, can reach to more students through instruction” |
|  |  |  | “Self-defense. If you don't teach them as a group, you're going to end up teaching them one at a time” |
| Time | Teaching is (or is not) viewed as a time saver, efficient, or convenient | 38 | “can train more people more quickly” |
|  |  |  | “Too many one to one requests otherwise” |
| Demand | Teaching is (or is not) demanded or requested | 34 | “Demand is high and easier to meet with students as a group than one-on-one” |
|  |  |  | “The demand for more curated/specific instruction” |
| Expertise | Teaching is (or is not) a skill, area of expertise, or knowledge area of the librarian | 26 | “It is one of the points in the education process where learners are thoroughly engaged in serious searching, so it is an opportunity to make sure that they have that knowledge” |
|  |  |  | “We’re considered experts in searching” |
| Job Task | Teaching is (or is not) viewed as a mandatory part of the job as a librarian, a responsibility, or a requirement. | 9 | “By request from faculty, because it's part of my liaison role” |
|  |  |  | “Because I believe it's up to me to give these trainings” |
| Support | Teaching is (or is not) supported, staffed, or part of a formalized system. | 5 | “lack of staffing (reach as many learners as possible in one go)” |
| Desire | Teaching is (or is not) enjoyable, desirable, or satisfactory | 4 | “Job Satisfaction” |
| Value | Teaching does (or does not) demonstrate the value of the librarian | 3 | “Demonstrate the importance of experienced librarian participation in KS work” |
|  |  |  | “To prove efficiency of librarians and library services to the community” |
