# Supplementary material for: Exploring librarians' practices when teaching advanced searching for knowledge synthesis: results from an online survey
Source: J Med Libr Assoc. 2024 Jul 29;112(3):238–49. doi: 10.5195/jmla.2024.1870 (PMC11412128; doi:10.5195/jmla.2024.1870)
Supplement: Supplementary file 6 — Appendix F: Responses for “Other” Tools Used [file jmla-112-3-238-s06.docx]

## Appendix F: Responses to “Other” for Question: “In preparing for, delivering, or following up on group instructional sessions, how often do you use the following tools to teach comprehensive searching methods for KS?”

- Journal articles, PRISMA tools - perhaps considered handout/worksheet; LMS - learning management system; written reflection paper
- Encourage pre-reading of the support materials; provide the support materials in a cloud drive ahead of the teaching sessions.
- Quick exercises, e.g. * dividing them into small groups and let each group find all possible synonyms for a concept, then allowing them to compare the result * jeans OR glasses; using the group as a database, asking the participants to stand up if they fulfill a criterion expressed as a search query, and many others
- Online content, practical exercise (formative assignment), individual feedback
- We had to create on online resource during Lockdown so took teaching powerpoint and chuncked it up and created lots of short videos and then used these to create online resource that students could use at point of need - Can't live without it now.
- Yale MESH analyzer - tool
- group video tutorials to follow up on search strategy
- Students sometimes get homework which they are supposed to complete and return for grading.
- Have not tried this yet, but plan to in the future: Students conduct a sample search at the start of the session, and then again at the end of the session after learning different searching tips and techniques. See how the searches may have changed/evolved as a result of the learning derived from the session.
- Storytelling, the power of stories of affirmation and success transcend most lectures and self-pace learning... (and pictures)
- In the past I've had participants do an activity on their own and then discuss the activity as a group
- Have students read two reviews, one in which the search is adequately described and well done, one in which it isn't. They are asked to compare the strategies.
- Pre-assessment surveys/activities before training begins.
- Exercises (students have time during the workshop to work on their own search strategy for their project)
- I sometimes give students a jumble of search terms for a research question. Then get them to put the terms into a search structure. Usually this is via shared word doc. Then they conduct the search and have to share number of results.
- Scan for relevance and share one citation which they think is likely to answer the question.
- I don't know where this fits, but by far the most effective instruction has been to have a dozen students in a room where ALL of them are engaged in searching to support a review (so they have an immediate application of the skills). Then the class is run so that you work through each step of the search (define question, parse question, find synonyms, execute searches (learn database), export results) with the whole group, but each person applying their learning to their own question. Doesn't waste any of the students time on stuff they don't need (like gamification and pictures of kittens). These people are time-starved. All they want is content that applies to them.
- Sometimes I'll use worksheets to help them visualize what they're doing
