# Supplementary material for: Exploring librarians' practices when teaching advanced searching for knowledge synthesis: results from an online survey
Source: J Med Libr Assoc. 2024 Jul 29;112(3):238–49. doi: 10.5195/jmla.2024.1870 (PMC11412128; doi:10.5195/jmla.2024.1870)
Supplement: Supplementary file 7 — Appendix G: Table Illustrating the Frequency of Respondents Who Cover Different Topics, Sorted in Order of Most Frequent to Least [file jmla-112-3-238-s07.docx]

Appendix G: Table illustrating the frequency of respondents who cover different topics, sorted in order of most frequent to least.

| **Topic** | **% Covering topic** | **total (n) covering topic** |
| --- | --- | --- |
| Boolean logic | 100.00 | 56 |
| Database selection | 98.21 | 55 |
| Synonym generation | 98.21 | 55 |
| Controlled vocabulary (eg. MeSH, Emtree) | 98.21 | 55 |
| Executing a database search | 98.21 | 55 |
| Translating search strategies | 96.43 | 54 |
| Database syntax | 94.64 | 53 |
| Search documentation | 94.64 | 53 |
| Refining review question | 92.86 | 52 |
| Question formulas (eg. PICOTT, PCC, SPIDER) | 92.86 | 52 |
| Determining appropriate review type | 91.23 | 52 |
| Reporting guidelines (eg. PRISMA) | 91.23 | 52 |
| Conduct/methodological guidance (eg. Cochrane MECIR standards, JBI Manual) | 91.23 | 52 |
| Testing search terms | 91.07 | 51 |
| Grey literature | 91.07 | 51 |
| Citation management software (eg. Endnote, RefWorks) | 91.07 | 51 |
| Deduplication | 91.07 | 51 |
| Search filters | 91.07 | 51 |
| Clinical trial registries | 87.50 | 49 |
| Screening | 83.93 | 47 |
| Sensitivity vs. precision | 82.14 | 46 |
| Evidence-based medicine | 80.36 | 45 |
| Protocol creation | 80.36 | 45 |
| Systematic review management software (eg. DistillerSR, Covidence) | 78.57 | 44 |
| Hierarchy of evidence | 70.18 | 40 |
| Critical appraisal | 67.86 | 38 |
| Reporting bias | 62.50 | 35 |
| Data extraction | 58.93 | 33 |
| Journal submission requirements | 55.36 | 31 |
| Test sets | 51.79 | 29 |
| Quantitative analysis/meta-analysis of results | 41.07 | 23 |
| Qualitative analysis of results | 35.09 | 20 |
